# Annual acknowledgement of manuscript reviewers

**DOI:** 10.1186/1471-230X-13-24

**Published:** 2013-03-07

**Authors:** Christopher Foote

**Affiliations:** 1 BMC Series, BioMed Central, 236 Grays Inn Road, London WC1X 8HB, UK

## Abstract

**Contributing reviewers:**

The editors of BMC Gastroenterology would like to thank all of the reviewers who contributed their time and efforts to the journal in volume 12 (2012).

## 

Raquel Abalo

Spain

Sohail Muhammad Afzal

France

Kosh Agarwal

UK

Alessio Aghemo

Italy

Jorgen Agnholt

Denmark

Massimo Agostoni

Italy

Irfan Ahmad

Sweden

Sang Hoon Ahn

South Korea

Tomohiko Akahoshi

Japan

Yasutada Akiba

USA

Farida Al Balushi

Oman

Luca antonio Aldrighetti

Italy

David Alpers

USA

Fernando Alvarez

Canada

Suresh Ambudkar

USA

Samad Amini-Bavil-Olyaee

USA

Basil Ammori

UK

Devendra Amre

Canada

Ashwin Ananthakrishnan

USA

Jeffery Anderson

USA

Frederic Andre

France

Montserrat Andreu

Spain

Tiing Leong Ang

Singapore

Beate Appenrodt

Germany

Jithesh Appukutty

UK

Richard Harry Argent

UK

Yoshiaki Arimura

Japan

Antonio Ascione

Italy

Ashish Atreja

USA

Christer Baeck

Germany

Matthias J. Bahr

Germany

Julio Bai

Argentina

Mona Bajaj-Elliott

UK

Young-Tae Bak

South Korea

Maria Elisabetta Baldassarre

Italy

Ludovic Barault

USA

Savio Barreto

India

Jacqueline Barrett

Australia

Daniel Barry

USA

Jyotsna Batra

Australia

Martijn Bauer

Netherlands

Hassan Baydoun

Lebanon

Lars Bechmann

Germany

Thomas Becker

Germany

Karim Bellagha

Tunisia

Derrick Bennett

UK

David Bernardo Ordiz

UK

Charles Bernstein

Canada

Nigel Bird

UK

Andrew Blann

UK

Kathleen Blondeau

Belgium

Mark Bloomston

USA

Dimitrios Bogdanos

UK

Gabriele Böhm

Germany

Josh Bomser

USA

Bruno Bonaz

France

Ole Bonderup

Denmark

Jan Bornschein

Germany

Xavier Bossuyt

Belgium

Mohamed Bourdi

USA

Alex Boussioutas

Australia

Federico Bozzetti

Italy

Barbara Braden

UK

Michael Bramble

UK

Albert Bredenoord

Netherlands

Axel Brehmer

Germany

Brian Bressler

Canada

Tim Bright

Australia

Allen Brinker

USA

Christina Brock

Denmark

PJ Brooks

USA

Tony Bruns

UK

Tony Bruns

Germany

Christophe Burucoa

France

Maria Antonella Burza

Sweden

Yilmaz Cakaloglu

Turkey

Ali Canbay

Germany

Guangwen Cao

China

Raffaele Capasso

Italy

Gabriele Capurso

Italy

Ross Carter

UK

Giulia Martina Cavestro

Italy

Christine Chambers

Canada

Walter Chan

USA

Chen-Wang Chang Chang

Taiwan

Rupesh Chaturvedi

USA

Liping Chen

China

Chien-Lin Chen

Taiwan

Juei-Tang Cheng

Taiwan

Cheng Tang Chiu

Taiwan

Seok-Reyol Choi

South Korea

Pao-Hsien Chu

Taiwan

Jody Church

Australia

Nicholas Church

UK

Edward Ciaccio

USA

Halina Cichoz-Lach

Poland

Paul Ciclitira

UK

Mihai Ciocirlan

Romania

John Clancy

USA

Lori Coburn

USA

Barbara Coco

Italy

Moïse Coëffier

France

Brad Confer

USA

Helena Cortez-Pinto

Portugal

Todd Costantini

USA

Charles Cox

USA

Filippo Cremonini

USA

Francisco Javier Cubero

Germany

Alessandro Cucchetti

Italy

Gloria Millaray Curilem

Chile

Olivier Cuvillier

France

Uta Dahmen

Germany

Francesco D'Amico

Italy

Srinivasan Dasarathy

USA

Severin Daum

Germany

Christopher Davis

USA

Rajinder Dawra

USA

Lukejohn Day

USA

Nicola de Bortoli

Italy

Colin de Haar

Netherlands

Aline de Piano

Brazil

Arjuna de silva

Sri Lanka

Thomas de Wijkerslooth

Netherlands

Ihsan Ekin Demir

Germany

Dajun Deng

China

Terry Derks

Netherlands

Marco DiBonaventura

USA

Ram Dickman

Israel

Levinus Dieleman

Canada

John Dillon

UK

Wen-Xing Ding

USA

Neil Gerard Docherty

Ireland

Laurent Dollé

Belgium

Claire Louise Donohoe

Ireland

Jennifer Drahos

USA

Donald Duerksen

Canada

Marjolijn Duijvestein

Netherlands

Chandradhar Dwivedi

USA

Eli Ehrenpreis

USA

J.J. Eloranta

Switzerland

Össur Ingi Emilsson

Iceland

Yuka Endo

Japan

Osman Ersoy

Turkey

Tuba Esfandyari

USA

Guy Eslick

Australia

Magdalena Esteva

Spain

Emilie Estrabaud

France

Evangelos Evangelou

Greece

Jonathan Evans

UK

Jian-gao Fan

China

Jia Fan

China

Fabio Farinati

Italy

Ricard Farre

Belgium

Per G Farup

Norway

Matteo Fassan

Italy

Andrew Feber

UK

Richelle Jeannette Fyline Felt

Netherlands

Bo Feng

China

Virginia Fernández

Chile

Marc Ferrante

Belgium

Bernd Fiebich

Germany

Christian Fingas

Germany

Lucia Fini

Italy

P. Marco Fisichella

USA

David Fleisher

USA

Ioannis Flessas

Greece

Antonio Fontanellas

Spain

Fernando Fornari

Brazil

Andrew Fraser

UK

Neal Freedman

USA

Martin Freeman

USA

Jeremy French

UK

Michael Fried

Switzerland

Serah Funke

Nigeria

Manuele Furnari

Italy

Pietro Fusaroli

Italy

Margherita Gallicchio

Italy

Julio Galvez

Spain

T. Clark Gamblin

USA

Chandrashekhar Gandhi

USA

Xin Gao

China

Pilar Garcia-Peñarrubia

Spain

Antonio Gasbarrini

Italy

Andreas Geier

Germany

Paula Ghaneh

UK

Lydia Giannitrapani

Italy

Alain Gobert

USA

Begoña González Suárez

Spain

Maria Francisca Gonzalez-Escribano

Spain

Stuart Gordon

USA

Ioannis Goulis

Greece

Guillaume Gourcerol

France

David Graham

USA

Roberto Grassi

Italy

Peter Green

USA

Thomas Griesbacher

Austria

Sergei Grivennikov

USA

Christophe Grosset

France

Annett Guenther

Germany

Antanas Gulbinas

Lithuania

Felix Gundling

Germany

Christina Ha

USA

Nicolai Haase

Denmark

Paul Haber

Australia

Panayiotis Hadjicostas

Cyprus

Ki-Baik Hahm

South Korea

Smita Halder

Canada

Christopher Halloran

UK

Zaed Hamady

UK

Jochen Hampe

Germany

Wei Han

China

Masayasu Hara

Japan

Werner Hartwig

Germany

Manal Hassan

USA

Goran Hauser

Croatia

Barney Hawthorne

UK

Michihiro Hayashi

Japan

Johannes Haybaeck

Austria

Matthew Hayden

USA

Refaat Hegazi

USA

Kerstin Herzer

Germany

Felix Heymann

Germany

Toshifumi Hibi

Japan

Kazuhide Higuchi

Japan

Michelle Hill

Australia

Gideon Hirschfield

Canada

Asahi Hishida

Japan

Megan Hitchins

Australia

Yuan-Soon Ho

Taiwan

Michael Hocke

Germany

Christoph Hoegenauer

Austria

Dieter Hoelzel

Germany

Joerg Hoffmann

Germany

Georgina Hold

UK

Milan Holecek

Czech Republic

Pamela Hornby

USA

Jason Hornick

USA

Jason Hou

USA

Fenghong Huang

China

Tien-Yu Huang

Taiwan

Jeffrey T.-J. Huang

UK

Martin Hug

Germany

Pali Hungin

UK

Keun Hur

USA

Steffen Husby

Denmark

Damian Hussey

Australia

Gyu-Sam Hwang

South Korea

Muhammad Idrees

Pakistan

Hiroaki Ikematsu

Japan

Hiroyuki Imaeda

Japan

Shinichi Imafuku

Japan

Ingmar Ipach

Germany

Dawn Israel

USA

Lene Hjerrild Iversen

Denmark

Junichi Iwamoto

Japan

Jakob Izbicki

Germany

Jeong Won Jahng

South Korea

Brendan Jenkins

Australia

Lars Henrik Jensen

Denmark

Ung Bae Jeon

South Korea

Christoph Jochum

Germany

Jon Gunnlaugur Jonasson

Iceland

Trine Lembrecht Jorgensen

Denmark

Hsueh-Fen Juan

Taiwan

Wolfgang Jugner

USA

Maciej Kaczmarski

Poland

Masaki Kaibori

Japan

Evangelos Kalaitzakis

Sweden

John Kalbfleisch

USA

Marinos Kallikourdis

Italy

Terumi Kamisawa

Japan

Ellen Kampman

Netherlands

Arne Kandulski

Germany

Dae Hwan Kang

South Korea

Jia-Horng Kao

Taiwan

Dimitrios Kapetanos

Greece

Georgios Karamanolis

Greece

John Katsimpris

Greece

Ishibashi Keiichiro

Japan

Paul Kelly

UK

Satish Keshav

UK

Alireza Keshtgar

UK

Mahmoud Khattab

Egypt

Reuben Kim

USA

Joseph Kim

USA

Myung-Hwan Kim

South Korea

Yoshikazu Kinoshita

Japan

Johannes Kluwe

Germany

Koichi Kobayashi

USA

Maurizio Koch

Italy

Arjun Koch

Netherlands

Robert Koch

Austria

Rakesh Kochhar

India

Gou Young Koh

South Korea

Tomas Koller

Slovakia

Orpheus Kolokythas

USA

Lars Komorowski

Germany

Hon Wai Koon

USA

Claus Kordes

Germany

Ilma Korponay-Szabo

Hungary

Richard Kozarek

USA

Kausilia Krishnadath

Netherlands

Lasse Sommer Kristensen

Denmark

Bernd Kronenberger

Germany

Alfreda Krupoves

Canada

Chao-Hung Kuo

Taiwan

Juozas Kupcinskas

Lithuania

Miloslav Kverka

Czech Republic

Slawomir Kwiecien

Poland

Jeffrey Lackner

USA

José M. Ladero

Spain

Peter Lakatos

Hungary

Stephen Lanspa

USA

Alberto Lanzini

Italy

Mike Larvin

UK

Joel Lavine

USA

Benjamin Lebwohl

USA

Hsuan-Shu Lee

Taiwan

Tommy Lee

USA

Grainne Lennon

Ireland

Andreas Leodolter

Germany

James Leonard

UK

Rupert Leong

Australia

Patrick Leung

USA

Yvette Leung

Canada

Rona Levy

USA

Yue Li

USA

Jieshou Li

China

Ying Li

Hong Kong

Christian Liedtke

Germany

Richard Lilford

UK

Hsi-Hsien Lin

Taiwan

Greger Lindberg

Sweden

Mikael Lindström

Sweden

Alexander Link

Germany

Iris Liou

USA

Ana Lleo

Italy

Amedeo Lonardo

Italy

Reginald V. Lord

Australia

Carmelo Luigiano

Italy

Veronika Lukacs-Kornek

USA

Holger Lutz

Germany

Pat Lynch

USA

Daqing Ma

UK

Fabio Salvatore Macaluso

Italy

Ryan Madanick

USA

Sanjiv Mahadeva

Malaysia

Salah Mahmud

Canada

Marcello Fabio Maida

Italy

Ali Majeed

UK

Mariano Malaguarnera

Italy

Georgia Malamut

France

Marco Manfredi

Italy

Elizabeth Mann

UK

Hendrik Manner

Germany

Julian Marchesi

UK

Vicente Martinez

Spain

Leonardo Marzio

Italy

Philippe Mathurin

France

Takahisa Matsuda

Japan

Dimitrios Matthaiou

Greece

Odike Maxy

Nigeria

George Mayne

Australia

Denis McCarthy

USA

Kenneth E.L. McColl

UK

Fermin Mearin

Spain

Valentina Medici

USA

Francis Megraud

France

Gerrit Meijer

Netherlands

Espen Melum

Norway

Emanuele Meroni

Italy

Neil Merrett

Australia

Marty Meyer

USA

Pal Miheller

Hungary

Bryan Miller

Canada

Michael Miller

UK

Masashi Mizokami

Japan

Lars Moeller

Germany

Martha Mohler

USA

Michele Molinari

Canada

Jong Ho Moon

South Korea

Leticia Moreira

Spain

Hisataka Moriwaki

Japan

Alexander Moschen

UK

Michael Moser

Canada

Maria Eugenia Motta

Brazil

Allan Mowat

UK

Marcus Muehlbauer

USA

Naoki Muguruma

Japan

Saurabh Mukewar

USA

Chris Mulder

Netherlands

Khalid Mumtaz

Canada

Jordi Muntané

Spain

Kazunari Murakami

Japan

Joseph Murray

USA

Pierre Nahon

France

Itaru Naitoh

Japan

Su Youn Nam

South Korea

Udayakumar Navaneethan

USA

Tomás Navarro-Rodriguez

Brazil

Rick Nelson

UK

Kristi Neufeld

USA

Manuela Neuman

Canada

Christoph Neumann-Haefelin

Germany

Jeremy Nightingale

UK

Mehrdad Nikfarjam

USA

Masashi Nishida

Japan

Markku Nissinen

Finland

Jerzy-Roch Nofer

Germany

Tsutomu Nomura

Japan

Fredrik Norström

Sweden

Gottfried Novacek

Austria

Federica Nuti

Italy

Ugochukwu Nzeako

USA

James O'Beirne

UK

Jude Oben

UK

Stefan Öberg

Sweden

Margarete Odenthal

Germany

Shuji Ogino

USA

Devin Oglesbee

USA

Dilara Ogunc

Turkey

Young Oh

USA

Lena Öhman

Sweden

Masayuki Ohtsuka

Japan

Mónica Oleastro

Portugal

Keith Chee Ooi

Australia

Derek O'Reilly

UK

William Orr

USA

Michiro Otaka

Japan

Katja Ott

Germany

Maggie Ow

UK

Fabio Pace

Italy

Mario Pages

Spain

Ferdinando Palmieri

Italy

Georgios Papachristou

USA

Basilios Papaziogas

Greece

Mária Papp

Hungary

Gisela Paul

Germany

Theodoros Pavlidis

Greece

Rishi Pawa

USA

Maria Pellise

Spain

Yen-Chun Peng

Taiwan

Marcello Persico

Italy

Mario Pescatori

Italy

Raffaele Pezzilli

Italy

Per Pfeiffer

Denmark

Thierry Piche

France

Oreste PIeramico

Italy

Guillaume Piessen

French Guiana

Mark Pimentel

USA

Fabio Piscaglia

Italy

Aurelie Plessier

France

Jan-Werner Poley

Netherlands

Alina Popescu

Romania

Chad Porter

USA

Mahmoudreza Pourkarim

Belgium

Dimitri Pournaras

UK

Vitaliy Poylin

USA

Muriel Priault

France

Massimo Primignani

Italy

Claudio Puoti

Italy

Nian-Song Qian

China

Andrew Quest

Chile

Enrique Quintero

Spain

Spiro Raftopoulos

Australia

Shan Rajendra

Australia

Maria Ramos

Spain

Shimon Reif

Israel

Max Reinshagen

Germany

Rungsun Rerknimitr

Thailand

Thomas Resch

Austria

Ki-Jong Rhee

South Korea

Jon Marc Rhoads

USA

Luigi Ricciardiello

USA

Eirini Rigopoulou

Greece

Kevin Roarty

USA

Michael Robek

USA

Barbera Roberta

Italy

Christoph Roderburg

Germany

Kryssia Rodriguez

Italy

Francisco Rodriguez Moranta

Spain

Simon Rogers

UK

Stefano Romeo

Sweden

Yvonne Romero

USA

April Camilla Roslani

Malaysia

Kamran Rostami

UK

Gianluca Rotondano

Italy

Maria Roubelakis

Greece

Praveen Roy

USA

Alberto Rubio Tapia

USA

Clara Ruiz-Ponte

Spain

Richard Russell

UK

Andrew Ruszkiewicz

Australia

Piotr Rutkowski

Poland

Russell Ryan

USA

Stephen Ryder

UK

Rodolfo Sacco

Italy

Amanda Sainsbury-Salis

Australia

Takafumi Saito

Japan

Yutaka Saito

Japan

Yuji Sakai

Japan

Osamu Sakamoto

Japan

Pedro Salas

USA

Eduardo Salazar-Lindo

Peru

Baljinder Salh

Canada

Faouzi Saliba

France

Mohammad Salih

Pakistan

Sundeep Saluja

India

Silvia Salvatore

Italy

Alex Sánchez-Pla

Spain

David Sanders

UK

Fuat Saner

Germany

Angelo Sangiovanni

Italy

Andrea Sartore Bianchi

Italy

Yasushi Sasaki

Japan

Lawrence Saubermann

USA

Edoardo Savarino

Italy

Franco Scaldaferri

Italy

Mario Scartozzi

Italy

Serena Schippa

Italy

Joerg Schlaak

Germany

Barbara Schneider

USA

Meike Schneider

Germany

Eckart Schott

Germany

Thomas Schreiter

Germany

Jochen Schuld

Germany

Hans-Ulrich Schulz

Germany

Michael Schumacher

USA

Oliver Schwandner

Germany

Rodney J. Scott

Australia

Masataka Seike

Japan

Anna Selby

UK

Michael Selgrad

Germany

Carlo Selmi

USA

Marco Senzolo

Italy

Claudio Sette

Italy

Maida Sewitch

Canada

George Sgourakis

Greece

Ala Sharara

Lebanon

Bo Shen

USA

Ying-Hong Shi

China

Ruihua Shi

China

Tomohiko Shimatani

Japan

Yehuda Shoenfeld

Israel

Nadeem Siddiqui

Pakistan

Giuseppe Signoriello

Italy

Jocelyn Silvester

UK

Alice Simon

UK

Siddharth Singh

USA

Shree R. Singh

USA

Amar Singh

USA

Suzanne Skoog

USA

Walter Smalley

USA

James L Smith

USA

Vassilios Smyrniotis

Greece

Harry Sokol

France

Javier Sola Vera

Spain

Andrei Sommer

Germany

Bridget Southwell

Australia

Manon Spaander

Netherlands

Anshu Srivastava

India

Martin Stacey

UK

Antoni Stadnicki

Poland

Frauke Stanke

Germany

Alistair Stewart

New Zealand

Susan Stewart

USA

Charmaine Stewart

USA

Ryan Stidham

USA

Konrad Streetz

Germany

Pavel Strnad

Germany

Vicki Strugala

UK

Abid Suddle

UK

K-C Sudhamshu

India

Wei-Zen Sun

Taiwan

Young-Joon Surh

South Korea

Robert Sutcliffe

UK

Svenja Sydor

Germany

Frank Tacke

Germany

Yoshitsugu Tajima

Japan

Tomohisa Takagi

Japan

Atsushi Takagi

Japan

Hirokazu Takahashi

Japan

Tetsuo Takehara

Japan

Yoji Takeuchi

Japan

Naping Tang

China

Tetsuya Tanigawa

Japan

Alessandra Tavani

Italy

Daniel Teitelbaum

USA

Theresia Thalhammer

Austria

Robert Thimme

Germany

Paul timpson

Australia

Yasuhiko Tomita

Japan

Jonel Trebicka

Germany

Eric Trepo

Belgium

Nicolas Tsavaris

Greece

Tomoya Tsukada

Japan

Kazuhiro Tsukamoto

Japan

Guido Tytgat

Netherlands

Norihisa Uehara

Japan

Ekrem Unal

Turkey

Daniel Urbain

Belgium

Hirofumi Uto

Japan

Efsevia Vakiani

USA

Filipa Vale

Portugal

Andre van Gossum

Belgium

Leo van Grunsven

Belgium

Anna van Nistelrooij

Netherlands

Hans van Rostenberghe

Malaysia

Hjalmar van Santvoort

Netherlands

Tom van Wezel

Netherlands

Gauri Varadhachary

USA

Francis Vasseur

France

Loredana Vecchione

Belgium

Marcelo F. Vela

USA

Sander Veldhuyzen van Zanten

Canada

Neve Vendt

Estonia

Cornelis Verhoef

Netherlands

G. Nicholas Verne

USA

Christopher Vickers

Australia

Silvia Vidal

Spain

Michael Vieth

Germany

Yana Vinogradova

UK

Santiago Vivas

Spain

Arndt Vogel

Germany

Umberto Volta

Italy

Stefan von Delius

Germany

Jakob Wallden

Sweden

Julian Walters

UK

Natasha Walzer

USA

Lisheng Wang

China

Yang Wang

USA

Yue Wang

China

Chrong-Reen Wang

Taiwan

Junping Wang

China

Yinghong Wang

USA

Huamin Wang

USA

Hsiu-Po Wang

Taiwan

Yinghong Wang

USA

Sachin Wani

USA

Sharon Weber

USA

Simon Wessely

UK

Thomas Wex

Germany

David Whiteman

Australia

Bas Wijnhoven

Netherlands

Liz Williams

UK

Eytan Wine

Canada

Kacper Wojtal

Switzerland

Banny Wong

USA

Grace Wong

Hong Kong

Alexander Wree

USA

Xianrui Wu

China

Karsten Wursthorn

Germany

Morten Würtz

Denmark

Kang Xiaonan

China

Lishou Xiong

China

Weining Xiong

China

Ling Xu

China

Patrick Yachimski

USA

Satheesh Yalamarthi

UK

Katsumi Yamamoto

Japan

Takahiro Yamasaki

Japan

Bo Yan

USA

Fang Yan

USA

Akinori Yanaka

Japan

Ning Yang

USA

Rhonda Yantiss

USA

Masakazu Yashiro

Japan

Kohichiroh Yasui

Japan

Shui Ye

USA

Andrew Yeoman

UK

Hiroshi Yoshida

Japan

Norimasa Yoshida

Japan

Howard Young

USA

Alex Zaika

USA

Petros Zezos

Greece

Yuan Zhai

USA

Guoxin Zhang

China

Minhua Zheng

China

Weiming Zhu

China

Bogna Ziarkiewicz-Wroblewska

Poland

Vincent Zimmer

Germany

Henning Zimmermann

Germany

Inti Zlobec

Switzerland

Oded Zmora

Israel

Angelo Zullo

Italy

## Pre-publication history

The pre-publication history for this paper can be accessed here:

http://www.biomedcentral.com/1471-230X/13/24/prepub

